# Clinical characteristics and prognostic factors in non-HIV patients with *Pneumocystis jirovecii* pneumonia and BALF cytomegalovirus co-detection: a retrospective cohort study

**DOI:** 10.3389/fpubh.2026.1921801

**Published:** 2026-08-26

**Authors:** Chaomin Guo, Yajiao An, Jingyun Ye, Yanning Ma, Qiang Zhao, Lifeng Wang, Wei Ma, Mingze Zhang, Miaoyu Wang, Yang Wang, Jiyong Yang, Liyan Ye, Liuyang Yang

**Affiliations:** 1 Laboratory Medicine Department, The First Medical Center, Chinese PLA General Hospital, Beijing, China; 2 Laboratory Medicine Department, Cengong County People's Hospital, Guizhou, China; 3 Department of Gynaecology and Obstetrics, The Second Affiliated Hospital of Chongqing Medical University, Chongqing, China; 4 Department of Pulmonary and Critical Care Medicine, The Second Medical Center and National Clinical Research Center for Geriatric Diseases, Chinese PLA General Hospital, Beijing, China; 5 Laboratory Medicine Department, Hainan Branch, Chinese PLA General Hospital, Sanya, Hainan, China

**Keywords:** co-detection, cytomegalovirus, metagenomic next-generation sequencing, non-HIV immunocompromised patients, *Pneumocystis jirovecii* pneumonia, prognostic biomarkers, retrospective cohort study

## Abstract

**Background:**

*Pneumocystis jirovecii* pneumonia (PJP) is a life-threatening opportunistic infection in immunocompromised patients. BALF cytomegalovirus (CMV) co-detection is frequently observed in PJP patients, but its clinical characteristics and prognostic impact in non-HIV populations remain unclear.

**Methods:**

In this single-center retrospective cohort study, we enrolled 62 non-HIV patients with confirmed PJP between 2019 and 2023. BALF CMV co-detection was defined as detectable CMV DNA in bronchoalveolar lavage fluid via metagenomic next-generation sequencing (mNGS), combined with compatible respiratory symptoms and chest computed tomography abnormalities. The Benjamini–Hochberg false discovery rate (FDR) correction was applied for multiple comparisons.

**Results:**

Overall, 31 patients (50.0%) had BALF CMV co-detection. The 28-day all-cause mortality was significantly higher in the CMV co-detection group than in the PJP-only group (54.84% vs. 19.35%, *p* = 0.008), and dyspnea was more prevalent (*p* = 0.024). After FDR correction for 39 laboratory parameters, only fibrinogen remained significantly lower in the co-detection group (*q* = 0.039), while (1,3)-β-D-glucan (BDG) and D-dimer showed independent associations with CMV co-detection in multivariable analyses. mNGS revealed more concurrent viral and fungal pathogens in the co-detection group, and multiple co-pathogens were more frequent in non-survivors within this subgroup. Interleukin-6 (IL-6), procalcitonin (PCT) and D-dimer were identified as independent prognostic factors for 28-day mortality in the CMV co-detection subgroup.

**Conclusion:**

In this single-center retrospective cohort, BALF CMV co-detection is associated with substantially elevated unadjusted 28-day mortality in non-HIV patients with PJP. These findings are hypothesis-generating; IL-6, PCT and D-dimer show potential as exploratory prognostic markers. Comprehensive mNGS-based pathogen screening combined with biomarker monitoring may facilitate risk stratification in this high-risk population, pending validation in larger prospective cohorts.

## 1 Introduction

*Pneumocystis jirovecii* pneumonia (PJP) is a life-threatening opportunistic fungal infection (1). Although PJP has traditionally been associated with HIV-infected individuals, it is increasingly diagnosed in non-HIV-infected patients, with hospital mortality rates ranging from 30% to over 60% (2, 3). An international multicenter retrospective analysis demonstrated that the majority of PJP cases in the intensive care unit (ICU) now occur in non-HIV-infected patients (3).

Among the infectious complications in immunocompromised patients, BALF cytomegalovirus (CMV) co-detection is of particular concern. CMV is frequently detected in the respiratory tract of immunocompromised individuals and commonly co-occurs with PJP (4). The reported prevalence of CMV co-detection among HIV-infected patients with PJP ranges from 28 to 69% (5); however, data on BALF CMV co-detection in non-HIV PJP patients remain scarce. Notably, CMV can be detected in the respiratory secretions of individuals without overt pneumonia symptoms, and the clinical significance of CMV detection—whether representing true tissue-invasive disease, viral reactivation, or colonization—in the context of PJP has not been systematically evaluated (6). Furthermore, the laboratory derangements, pathogen landscape, and prognostic factors associated with PJP with BALF CMV co-detection in non-HIV populations remain poorly characterized. Metagenomic next-generation sequencing (mNGS) of bronchoalveolar lavage fluid (BALF) enables unbiased, comprehensive pathogen detection and may help delineate the co-infecting microbial profile in this population (7, 8).

In this study, we analyzed a retrospective cohort of 62 non-HIV PJP patients who underwent BALF mNGS to characterize the clinical characteristics and prognostic factors associated with PJP and BALF CMV co-detection. To our knowledge, this is among the first studies to characterize the clinical and prognostic profiles of PJP with BALF CMV co-detection via metagenomic next-generation sequencing in a non-HIV cohort, and the first to apply Benjamini-Hochberg FDR correction for multiple laboratory comparisons in this context. We hypothesized that BALF CMV co-detection is associated with distinct laboratory derangements and worse clinical outcomes in non-HIV PJP patients.

## 2 Patients and methods

### 2.1 Study design and patients

This was a single-center retrospective cohort study. We conducted a retrospective analysis of 62 non-HIV patients diagnosed with PJP based on positive Gomori methenamine silver (GMS) staining of BALF and consistent clinical presentation at the First Medical Center of PLA General Hospital between January 2019 and December 2023. Patients were consecutively enrolled, with a relatively balanced case distribution across the 5-year study period. All patients diagnosed with PJP during the study period were included; no additional exclusion criteria were applied. No formal sample size calculation was performed. All patients underwent bronchoalveolar lavage fluid (BALF) collection using a flexible bronchoscope and standard procedures. The use of immunosuppressive agents combined with systemic glucocorticoids was defined as patients receiving at least one class of immunosuppressant (calcineurin inhibitors, antimetabolites, mTOR inhibitors, targeted biologics or chemotherapy) plus systemic prednisone-equivalent glucocorticoids ≥10 mg daily for ≥2 consecutive weeks within 3 months prior to PJP onset. All patients underwent routine HIV antibody screening via enzyme-linked immunosorbent assay (ELISA) within 24 h of hospital admission, and all tested negative with no clinical history of HIV infection, thereby confirming non-HIV status.

### 2.2 Diagnostic criteria

All included patients were diagnosed with PJP based on positive Gomori methenamine silver (GMS) staining of BALF combined with consistent clinical and radiological presentation. Quantitative PCR for *Pneumocystis jirovecii* load in BALF was not routinely performed in clinical practice, and thus fungal burden data were not available for this cohort. BALF CMV co-detection was defined as detectable CMV DNA sequences in BALF via mNGS, together with compatible clinical manifestations (fever, cough, dyspnea or other respiratory symptoms) and pulmonary CT findings (ground-glass opacities, interstitial infiltration or other abnormalities consistent with viral pneumonia).

### 2.3 Data collection

Clinical data, including laboratory results obtained within 3 days of PJP diagnosis, were extracted from the electronic medical record system.

### 2.4 mNGS of bronchoalveolar lavage fluid (BALF)

Bronchoalveolar lavage fluid samples were subjected to DNA mNGS and/or RNA mNGS following established protocols (9). DNA was extracted using a QIAamp UCP Pathogen DNA Kit (Qiagen) (10). Total RNA was extracted with a QIAamp Viral RNA Kit (Qiagen), depleted of ribosomal RNA, and reverse-transcribed into cDNA (11). DNA and cDNA libraries were constructed using a Nextera XT DNA Library Prep Kit (Illumina) and sequenced on an Illumina NextseqCN500 platform. Negative and non-template controls were processed in parallel.

Raw reads were filtered with Trimmomatic to remove low-quality sequences, adapter contaminants, duplicates, and reads <50 bp; low-complexity reads were removed with Kcomplexity (12). Human reads were excluded by mapping to hg38 using Burrows-Wheeler Aligner (13). Remaining reads were classified taxonomically using Kraken2 against NCBI reference genomes (bacterial, archaeal, fungal, viral, invertebrate, and protozoan) and species-level abundance was quantified with Bracken (14). A positive detection was defined as Reads Per Million (RPM) ratio (RPM_sample/RPM_NTC) ≥ 10 for species with background in negative controls, or RPM ≥ 0.05 for species without background (11). Detailed sequencing parameters and quality control thresholds are provided in Supplementary Methods (15).

### 2.5 Ethical approval

This study was approved by the Institutional Review Board (IRB) of the First Medical Center of the General Hospital of the People’s Liberation Army (IRB number: S2024-411-01). All procedures were conducted in accordance with applicable regulations, standards and the Declaration of Helsinki. Due to the retrospective design of the study, the IRB granted a waiver for informed consent.

### 2.6 Statistical analyses

The primary endpoint was 28-day all-cause mortality, selected in accordance with standard conventions in critical care research for short-term prognostic evaluation. Secondary outcomes included laboratory abnormalities, chest imaging manifestations, and pathogen profiles identified via mNGS.

**Figure 1:**
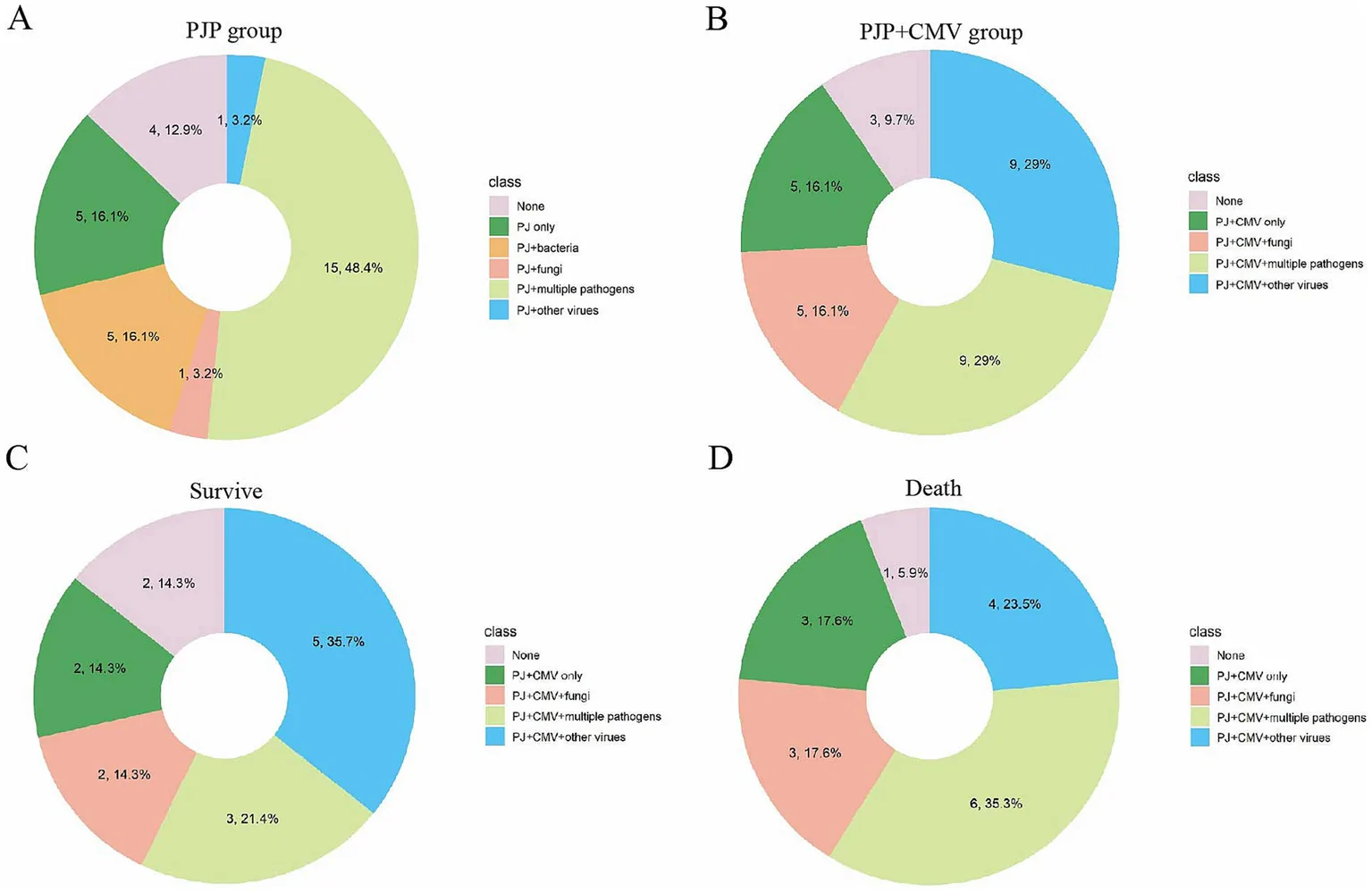
Proportions of co-pathogen categories detected by mNGS in the PJP group **(A)** and PJP + CMV group **(B)**, and in the survival **(C)** and mortality **(D)** subgroups within the PJP + CMV cohort. The category “None” indicates no additional pathogens detected beyond *Pneumocystis jirovecii* (and CMV in the PJP + CMV group).

Normality of continuous variables was assessed using the Shapiro–Wilk test. Normally distributed data were reported as mean ± SD and compared using independent samples *t*-tests; non-normally distributed data were presented as median (IQR) and analyzed with the Mann–Whitney *U* test. Categorical data were summarized as counts (percentages) and compared using the *χ*^2^ test; Fisher’s exact test was used when any expected cell count was < 5. Multivariate linear and logistic regression models were constructed to evaluate the independent association of BALF CMV co-detection with laboratory and imaging indicators: (1) Model 1: adjusted for age and sex; (2) Model 2: further adjusted for baseline comorbidities (diabetes, hypertension, chronic lung disease, hematological malignancy, rheumatic disease, solid tumor, glomerulonephritis/nephrosis); (3) Model 3: additionally adjusted for solid organ transplantation history and combined immunosuppressant plus glucocorticoid exposure. Solid organ transplantation was defined as prior kidney or liver transplantation with ongoing long-term maintenance immunosuppression. All skewed laboratory biomarkers underwent natural logarithmic transformation before regression to approximate a normal distribution. The *β* coefficient reflects the log-fold change in raw biomarker levels associated with BALF CMV co-detection (*β* ≈ 0.693 corresponds to a doubling of biomarker concentration). Hazard ratios (HR) derived from Cox models represent the relative mortality risk per e-fold elevation of log-transformed biomarkers. Cox proportional hazards regression was used to identify independent predictors of 28-day mortality in the CMV co-detection subgroup. Benjamini-Hochberg FDR correction was applied for multiple comparisons of laboratory and imaging parameters, with *q* < 0.05 defined as the threshold for statistical significance. Missing data were handled via complete case analysis; the proportion of missing data was less than 5% across all variables. Comparisons of microbial composition (Figure 1) were performed using the *χ*^2^ test or Fisher’s exact test as appropriate. All categorical comparisons were recalculated; *p* values in Tables 1, 2 and Figure 1 have been updated accordingly.

**Table 1:** Baseline and clinical characteristics of PJP and PJP combined with CMV patients.

Characteristic	Overall (*n* = 62)	PJP (*n* = 31)	PJP + CMV (*n* = 31)	*P*
Baseline characteristics				
Age, years, mean (SD)	54.89 (18.32)	54.74 (17.95)	55.03 (18.99)	0.951
Men, *n* (%)	40 (64.52)	19 (61.29)	21 (67.74)	0.791
Duration of hospitalization, mean (SD)	26.76 (17.50)	23.71 (15.54)	29.81 (19.04)	0.172
Immune agents combined with glucocorticoids use, *n* (%)	19 (30.65)	6 (19.35)	13 (41.94)	0.098
Clinical manifestations				
Dyspnea, *n* (%)	24 (39.34)	7 (22.58)	17 (54.84)	0.024
Comorbidities				
Diabetes, *n* (%)	15 (24.19)	7 (22.58)	8 (25.81)	1.000
Hypertension, *n* (%)	18 (29.03)	7 (22.58)	11 (35.48)	0.401
Chronic lung disease, *n* (%)	3 (4.84)	3 (9.68)	0 (0.00)	0.238
Organ transplantation, *n* (%)	8 (12.90)	1 (3.23)	7 (22.58)	0.053
Hematological malignant tumor, *n* (%)	10 (16.13)	4 (12.90)	6 (19.35)	0.732
Rheumatic disease, *n* (%)	7 (11.29)	6 (19.35)	1 (3.23)	0.104
Solid organ tumor, *n* (%)	6 (9.68)	2 (6.45)	4 (12.90)	0.671
Glomerulonephritis or nephrotic syndrome, *n* (%)	3 (4.84)	2 (6.45)	1 (3.23)	1.000
Organ support and invasive procedures				
Non-invasive ventilation, *n* (%)	2 (3.23)	2 (6.45)	0 (0.00)	0.492
Tracheal intubation, *n* (%)	14 (22.58)	4 (12.90)	10 (32.26)	0.127
Venous catheterization, *n* (%)	3 (4.84)	0 (0.00)	3 (9.68)	0.238
CRRT, *n* (%)	2 (3.23)	1 (3.23)	1 (3.23)	1.000
ECMO, *n* (%)	1 (1.61)	1 (3.23)	0 (0.00)	1.000
Clinical outcome				
28-day mortality, *n* (%)	23 (37.10)	6 (19.35)	17 (54.84)	0.008

**Table 2:** Laboratory parameters and pulmonary imaging features of PJP and PJP combined with CMV patients.

Parameter	PJP (*n* = 31)	PJP + CMV (*n* = 31)	*P*	FDR-adjusted *q* value
Laboratory parameters, median (IQR)				
BDG (ng/L)	**157.50 (22.65, 317.05)**	**404.25 (177.30, 508.50)**	**0.003**	0.0585
WBC (×10^9^/L)	7.92 (5.50, 9.58)	5.77 (3.97, 8.69)	0.215	0.4738
NEU (%)	0.80 (0.69, 0.89)	0.88 (0.75, 0.92)	0.331	0.5613
PaO_2_ (mmHg)	71.95 (59.50, 95.78)	69.60 (63.55, 98.32)	0.769	0.9518
PaCO_2_ (mmHg)	35.95 (31.33, 39.88)	34.80 (32.57, 40.93)	0.729	0.9518
PaO_2_/PaCO_2_	1.92 (1.72, 2.67)	1.96 (1.71, 2.83)	0.716	0.9518
LDH (U/L)	**303.40 (233.75, 388.45)**	**438.50 (321.60, 648.75)**	**0.013**	0.1690
CRP (mg/L)	2.64 (1.26, 8.94)	6.14 (0.96, 8.62)	0.751	0.9518
IL-6 (pg/mL)	10.59 (3.52, 74.89)	17.52 (3.28, 62.53)	0.965	1.0000
PCT (ng/mL)	0.16 (0.06, 0.47)	0.18 (0.07, 1.10)	0.243	0.4738
NT-proBNP (pg/mL)	381.25 (81.82, 992.60)	708.20 (275.82, 1625.75)	0.147	0.4738
LYM (/μl)	764.66 (337.42, 1222.21)	395.04 (195.84, 1042.86)	0.061	0.2974
LYM-T (/μl)	**602.92 (275.59, 1044.76)**	**208.24 (149.63, 654.67)**	**0.032**	0.2080
LYM-B (/μl)	35.61 (0.79, 178.57)	30.77 (9.23, 73.63)	0.781	0.9518
CD4^+^T (/μl)	176.99 (110.47, 371.37)	120.28 (29.25, 315.60)	0.151	0.4738
CD8^+^T (/μl)	279.65 (95.00, 475.89)	119.50 (73.10, 282.82)	0.236	0.4738
CD3^+^T (%)	71.04 (55.36, 80.75)	71.37 (64.19, 90.68)	0.229	0.4738
CD4^+^/CD3^+^ (%)	**36.92 (25.77, 46.76)**	**26.25 (17.09, 38.95)**	**0.046**	0.2563
CD8^+^/CD3^+^ (%)	29.76 (21.40, 42.77)	35.21 (26.64, 66.84)	0.106	0.4593
CD4^+^/CD8^+^ (%)	1.05 (0.64, 1.94)	0.67 (0.31, 1.48)	0.132	0.4738
TT (s)	16.50 (15.95, 19.05)	16.80 (15.90, 18.90)	0.966	1.0000
APTT (s)	38.90 (32.00, 45.60)	35.40 (30.55, 40.45)	0.234	0.4738
PT (s)	14.00 (13.55, 14.80)	14.20 (13.20, 15.85)	0.564	0.8798
PTA (%)	85.00 (78.00, 91.50)	83.60 (66.50, 97.00)	0.627	0.9201
INR	1.08 (1.04, 1.16)	1.10 (1.02, 1.29)	0.637	0.9201
FIB (g/L)	**4.35 (3.55, 5.54)**	**2.90 (2.56, 4.23)**	**0.001**	0.0390*
D-dimer (mg/L)	**0.84 (0.36, 2.01)**	**1.80 (0.84, 5.26)**	**0.030**	0.2080
Pulmonary imaging features, *n* (%)				
Ground-glass opacity	17 (54.84)	12 (38.71)	0.309	0.5478
Other high-density opacities	17 (54.84)	21 (67.74)	0.434	0.7052
Strands or band-like shadows	12 (38.71)	3 (9.68)	0.018	0.1755
Nodules	18 (58.06)	12 (38.71)	0.204	0.4738
Consolidation	6 (19.35)	6 (19.35)	1	1.0000
Pulmonary bullae	5 (16.13)	4 (12.90)	1	1.0000
Cavities	1 (3.23)	5 (16.13)	0.198	0.4738
Fibrosis	2 (6.45)	1 (3.23)	1	1.0000
Bronchial wall thickening	3 (9.68)	2 (6.45)	1	1.0000
Pleural effusion	12 (38.71)	17 (54.84)	0.309	0.5478
Hydropneumothorax	0 (0.00)	1 (3.23)	1	1.0000
Pleural thickening	3 (9.68)	0 (0.00)	0.237	0.4738

BDG is reported in ng/L, which is numerically equivalent to pg/mL. PTA: prothrombin time activity, unit: %. LYM-T: total T lymphocyte counts; LYM-B: B lymphocyte counts; CD4+/CD3+: CD4 + T cell percentage among CD3 + T cells; CD8+/CD3+: CD8 + T cell percentage among CD3 + T cells; CD4+/CD8+: CD4 + to CD8 + T cell ratio. Missing data proportion was <5% across all parameters, and complete case analysis was applied.

*q < 0.05 after Benjamini–Hochberg FDR correction for 39 comparisons.

Bold values indicate mean statistical significance (*P* < 0.05).

## 3 Results

### 3.1 Participant characteristics

A total of 62 patients with microbiologically confirmed PJP were included in this study, half of whom (31 cases, 50.0%) had BALF CMV co-detection (PJP with CMV co-detection group; Table 1). There were no statistically significant differences between the PJP-only group and the CMV co-detection group in terms of age, sex, duration of hospitalization, or receipt of non-invasive ventilation. Duration of hospitalization is presented for descriptive purposes only and may be confounded by early mortality, and thus is not interpreted as an indicator of disease severity. Of note, combined immunosuppressant plus glucocorticoid use (19.35% vs. 41.94%, *p* = 0.098) and history of solid organ transplantation (3.23% vs. 22.58%, *p* = 0.053) showed a trend toward higher prevalence in the CMV co-detection group with borderline statistical significance, which may not have reached the conventional significance threshold due to the limited sample size. Regarding clinical manifestations, the CMV co-detection group had a significantly higher prevalence of dyspnea than the PJP-only group (*p* = 0.024). No statistically significant differences were observed between the two groups in other baseline comorbidities, including diabetes, hypertension, chronic lung disease, hematological malignancies, rheumatic diseases, solid organ tumors, and glomerulonephritis/nephrotic syndrome. Importantly, the CMV co-detection group had a substantially higher 28-day all-cause mortality rate than the PJP-only group (54.84, 95% CI 37.74–70.79% vs. 19.35, 95% CI 9.19–36.29%; absolute risk difference 35.48, 95% CI 12.92–58.05%; *p* = 0.008; Table 1).

### 3.2 Laboratory parameters and pulmonary imaging features

We summarized the laboratory parameters obtained at the time of diagnosis for both the PJP group and the PJP + CMV group in Table 2. The PJP + CMV group showed nominal differences in laboratory parameters, including BDG, LDH, T cell counts, CD4+/CD3 + ratio, FIB, and D-dimer levels (all *p* < 0.05). After Benjamini-Hochberg FDR correction for 39 comparisons, only FIB remained statistically significant (FDR-adjusted *q* = 0.039). Of note, while BDG did not survive FDR correction in univariate analysis, its independent association with BALF CMV co-detection was confirmed in multivariate linear regression (*β* = 1.488, 95% CI 0.699–2.276, *p* = 0.001; Table 3).

**Table 3:** Multivariate linear and logistic regression analyses for the association of BALF CMV co-detection with laboratory parameters and pulmonary imaging features in PJP patients.

Parameter	Model 1		Model 2		Model 3	
	*β*/OR (95%CI)	*P*	*β*/OR (95%CI)	*P*	*β*/OR (95%CI)	*P*
Laboratory parameters						
BDG	**1.269 (0.588, 1.950)**	**0.001**	**1.153 (0.362, 1.944)**	**0.006**	**1.488 (0.699, 2.276)**	**0.001**
WBC	−0.117 (−0.409, 0.175)	0.435	−0.148 (−0.471, 0.176)	0.376	−0.122 (−0.471, 0.226)	0.495
NEU	0.038 (−0.060, 0.135)	0.449	0.029 (−0.077, 0.135)	0.593	0.043 (−0.071, 0.157)	0.461
PaO₂	0.012 (−0.160, 0.184)	0.893	0.029 (−0.172, 0.229)	0.781	−0.003 (−0.21, 0.203)	0.975
PaCO₂	−0.021 (−0.145, 0.104)	0.746	−0.053 (−0.197, 0.092)	0.477	−0.045 (−0.198, 0.107)	0.566
PaO_₂_/PaCO_₂_	0.033 (−0.188, 0.253)	0.774	0.081 (−0.171, 0.333)	0.53	0.042 (−0.216, 0.300)	0.753
LDH	0.291 (−0.069, 0.652)	0.119	0.233 (−0.179, 0.644)	0.273	0.220 (−0.225, 0.665)	0.338
CRP	0.104 (−0.771, 0.978)	0.817	−0.196 (−1.179, 0.787)	0.698	−0.070 (−1.111, 0.971)	0.896
IL-6	0.065 (−0.857, 0.987)	0.890	−0.099 (−1.106, 0.908)	0.847	0.147 (−0.825, 1.119)	0.768
PCT	0.609 (−0.441, 1.659)	0.261	0.224 (−0.961, 1.409)	0.713	0.226 (−1.038, 1.490)	0.728
NT-proBNP	0.967 (−0.087, 2.021)	0.077	0.882 (−0.295, 2.059)	0.148	0.998 (−0.164, 2.160)	0.099
LYM	**−0.622 (−1.201, −0.043)**	**0.041**	−0.380 (−1.025, 0.265)	0.256	−0.518 (−1.171, 0.136)	0.129
LYM-T	**−1.026 (−1.801, −0.252)**	**0.012**	**−0.975 (−1.872, −0.077)**	**0.039**	**−1.210 (−2.120, −0.299)**	**0.013**
LYM-B	−0.586 (−1.999, 0.828)	0.421	−0.122 (−1.590, 1.345)	0.871	−0.485 (−1.995, 1.024)	0.533
CD3+	0.495 (−0.137, 1.127)	0.131	**0.736 (0.074, 1.398)**	**0.035**	**0.797 (0.090, 1.504)**	**0.033**
CD4+	−0.448 (−1.433, 0.537)	0.377	0.004 (−1.002, 1.011)	0.993	−0.198 (−1.260, 0.863)	0.716
CD8+	−0.157 (−1.026, 0.711)	0.724	0.203 (−0.704, 1.110)	0.663	−0.026 (−0.972, 0.920)	0.958
CD4+/CD3+	−0.364 (−0.776, 0.047)	0.089	−0.401 (−0.866, 0.065)	0.099	−0.476 (−0.964, 0.012)	0.063
CD8+/CD3+	0.227 (−0.044, 0.497)	0.107	0.219 (−0.089, 0.527)	0.171	0.235 (−0.089, 0.560)	0.163
CD4+/CD8+	−0.458 (−1.015, 0.099)	0.113	−0.423 (−1.036, 0.190)	0.184	−0.508 (−1.150, 0.135)	0.129
TT	−0.092 (−0.290, 0.106)	0.367	−0.157 (−0.374, 0.059)	0.161	−0.197 (−0.429, 0.035)	0.103
APTT	−0.092 (−0.234, 0.050)	0.210	−0.132 (−0.292, 0.028)	0.111	−0.097 (−0.266, 0.072)	0.268
PT	0.008 (−0.060, 0.077)	0.811	−0.015 (−0.087, 0.058)	0.694	−0.005 (−0.083, 0.073)	0.901
PTA	0.017 (−0.144, 0.178)	0.836	0.044 (−0.131, 0.220)	0.622	0.048 (−0.140, 0.237)	0.617
INR	−0.100 (−0.389, 0.188)	0.498	−0.159 (−0.484, 0.166)	0.342	−0.220 (−0.564, 0.123)	0.214
FIB	**−0.346 (−0.544, −0.147)**	**0.001**	**−0.404 (−0.622, −0.186)**	**0.001**	**−0.395 (−0.631, −0.160)**	**0.002**
D-dimer	**0.745 (0.128, 1.363)**	**0.021**	**0.734 (0.041, 1.428)**	**0.043**	**0.831 (0.088, 1.573)**	**0.033**
Pulmonary imaging features						
Ground-glass opacity	0.515 (0.187, 1.422)	0.201	0.468 (0.165, 1.331)	0.155	0.485 (0.164, 1.439)	0.192
Other high-density opacities	1.723 (0.607, 4.890)	0.306	1.829 (0.628, 5.327)	0.269	1.516 (0.501, 4.588)	0.462
Strands or band-like shadows	**0.164 (0.039, 0.683)**	**0.013**	**0.158 (0.037, 0.674)**	**0.013**	**0.149 (0.033, 0.676)**	**0.014**
Nodules	0.445 (0.160, 1.239)	0.121	0.399 (0.138, 1.148)	0.088	0.373 (0.123, 1.132)	0.082
Consolidation	0.852 (0.219, 3.309)	0.817	0.899 (0.219, 3.695)	0.883	0.959 (0.226, 4.079)	0.955
Pulmonary bullae	0.670 (0.152, 2.948)	0.596	0.755 (0.160, 3.563)	0.723	0.573 (0.108, 3.033)	0.513
Fibrosis	5.574 (0.600, 51.783)	0.131	5.114 (0.467, 49.664)	0.995	5.064 (0.464, 47.745)	0.995
Bronchial wall thickening	0.390 (0.031, 4.854)	0.464	0.303 (0.023, 4.031)	0.366	0.175 (0.010, 3.188)	0.239
Pleural effusion	0.558 (0.082, 3.774)	0.549	1.792 (0.145, 22.137)	0.649	2.900 (0.210, 40.119)	0.427

Model 1: adjusted for age and sex; Model 2: additionally adjusted for history of diseases; Model 3: additionally adjusted for transplantation and immune agents combined with glucocorticoids use.

All continuous laboratory parameters were natural log-transformed prior to analysis. β coefficients are presented for linear regression of laboratory parameters; odds ratios (OR) are presented for logistic regression of imaging features.

Bold values indicate mean statistical significance (*P* < 0.05).

In terms of pulmonary imaging features, the PJP group showed a higher prevalence of strand-like or band-like shadows (38.71%) compared with the PJP + CMV group (9.68%; nominal *p* = 0.018), though this difference did not remain significant after FDR correction (*q* = 0.176).

### 3.3 Pathogen categories

In addition to PJP and CMV, we identified other co-detected microorganisms using mNGS (Figure 1). The PJP + CMV group exhibited a higher proportion of viruses (29.0% vs. 3.2%) and fungi (16.1% vs. 3.2%), but a lower proportion of bacteria (0% vs. 16.1%; Figures 1A,B). Specifically, *Epstein–Barr virus*, *Torque teno virus* and *Candida albicans* were the most common co-pathogens in both the PJP and PJP + CMV groups (Supplementary Table S1). Additionally, other opportunistic pathogens such as *Aspergillus* and *Mycobacterium* were also detected by mNGS (Supplementary Table S1). We also analyzed pathogen categories in the survival and mortality subgroups within the PJP + CMV group. The mortality subgroup had a higher proportion of multiple pathogens (35.3% vs. 21.4%) and a lower proportion of co-detected viruses (23.5% vs. 35.7%) compared with the survival subgroup, though these differences did not reach statistical significance (Figures 1C,D).

### 3.4 Multivariate linear regression analyses of laboratory parameters and pulmonary imaging features in PJP patients

After adjusting for multiple covariates, BDG levels, T lymphocyte subsets, FIB, and D-dimer were independently associated with PJP with BALF CMV co-detection (Table 3). Compared with the PJP group, the PJP + CMV group exhibited significantly elevated BDG levels (*β* = 1.488; 95% CI = 0.699, 2.276; *p* = 0.001), increased percentage of CD3 + T lymphocytes among total lymphocytes (*β* = 0.797; 95% CI = 0.090, 1.504; *p* = 0.033), and higher D-dimer levels (*β* = 0.831; 95% CI = 0.088, 1.573; *p* = 0.033). In contrast, absolute total T lymphocyte counts (*β* = −1.210; 95% CI = −2.120, −0.299; *p* = 0.013) and fibrinogen (FIB) levels (*β* = −0.395; 95% CI = −0.631, −0.160; *p* = 0.002) were significantly decreased in the PJP + CMV group. Additionally, patients with CMV co-detection had a decreased risk of developing strand-like or band-like shadows on imaging (OR = 0.149; 95% CI = 0.033, 0.676; *p* = 0.014).

### 3.5 Cox regression analysis of risk factors for 28-day mortality in the PJP with CMV co-detection subgroup

The PJP + CMV co-detection subgroup was divided into survivors and non-survivors according to 28-day all-cause mortality (Table 4). Statistically significant differences were observed in C-Reactive Protein, Interleukin (IL)-6, Procalcitonin (PCT), PT, PTA, INR, FIB, and D-dimer levels between the two subgroups (*p* < 0.05). The association between risk factors and survival or mortality events in the PJP + CMV group was determined using Cox regression analysis (Table 5). After adjustments in Models 2 and 3, IL-6, PCT, and D-dimer remained significantly associated with mortality events. In Model 3, the hazard ratios and 95% confidence intervals for IL-6, PCT, and D-dimer were as follows: IL-6 (HR = 1.915; 95% CI = 1.094, 3.352), PCT (HR = 1.642; 95% CI = 1.171, 2.302), and D-dimer (HR = 4.686; 95% CI = 1.529, 14.358). These findings indicate that IL-6, PCT and D-dimer were correlated with mortality in the PJP + CMV group in Models 2 and 3.

**Table 4:** Laboratory parameters and pulmonary imaging features stratified by 28-day mortality in the PJP combined with CMV group.

Parameter	Survive (*n* = 14)	Death (*n* = 17)	*P*
Laboratory parameters, median (IQR)			
BDG	414.30 (157.30, 514.60)	394.20 (293.20, 490.20)	0.522
WBC	5.68 (4.83, 7.21)	7.84 (3.81, 10.14)	0.341
NEU	0.88 (0.67, 0.93)	0.88 (0.76, 0.90)	0.843
PaO_2_	95.45 (66.40, 106.15)	65.60 (63.12, 78.07)	0.109
PaCO_2_	35.55 (31.30, 39.05)	34.15 (33.05, 44.65)	0.963
PaO_2_/PaCO_2_	2.61 (1.92, 3.00)	1.91 (1.63, 2.74)	0.164
LDH	403.85 (284.75, 566.25)	438.50 (375.60, 718.60)	0.475
CRP	**1.26 (0.66, 6.32)**	**7.30 (5.25, 10.28)**	**0.011**
IL-6	**6.04 (1.82, 22.17)**	**18.46 (9.46, 70.99)**	**0.035**
PCT	**0.07 (0.06, 0.19)**	**0.59 (0.17, 1.48)**	**0.009**
NT-proBNP	516.00 (226.20, 976.30)	892.40 (279.20, 1938.00)	0.336
LYM	422.35 (258.20, 1085.40)	367.73 (155.62, 764.96)	0.309
LYM-T	284.52 (163.73, 715.71)	197.51 (129.89, 638.79)	0.284
LYM-B	48.93 (29.41, 123.86)	23.18 (0.23, 50.91)	0.065
CD4+	137.00 (78.76, 315.60)	75.78 (24.36, 237.55)	0.325
CD8+	108.00 (73.10, 329.45)	122.62 (76.40, 214.42)	0.854
CD4+/CD3+	30.37 (25.16, 39.00)	18.98 (16.51, 35.14)	0.242
CD8+/CD3+	35.12 (26.35, 46.58)	44.26 (27.17, 70.80)	0.242
CD4+/CD8+	0.76 (0.46, 1.48)	0.45 (0.24, 1.36)	0.255
CD3+	69.87 (63.94, 77.66)	83.76 (64.58, 91.11)	0.325
TT	16.70 (16.00, 18.65)	16.80 (15.60, 18.90)	0.952
APTT	34.05 (26.97, 36.90)	38.20 (30.90, 44.10)	0.104
PT	**13.45 (12.93, 14.07)**	**15.40 (14.20, 16.30)**	**0.013**
PTA	**92.50 (84.45, 102.40)**	**71.00 (63.00, 84.00)**	**0.008**
INR	**1.02 (0.98, 1.09)**	**1.24 (1.10, 1.33)**	**0.007**
FIB	**2.79 (2.27, 2.89)**	**3.75 (2.83, 4.54)**	**0.022**
D-dimer	**0.84 (0.44, 1.62)**	**3.59 (2.28, 8.89)**	**<0.001**
Pulmonary imaging features, *n* (%)			
Ground-glass opacity	5 (35.71)	7 (41.18)	1
Other high-density opacities	10 (71.43)	11 (64.71)	0.990
Strands or band-like shadows	3 (21.43)	0 (0.00)	0.162
Nodules	8 (57.14)	4 (23.53)	0.123
Consolidation	2 (14.29)	4 (23.53)	0.848
Pulmonary bullae	3 (21.43)	1 (5.88)	0.455
Cavities	3 (21.43)	2 (11.76)	0.812
Fibrosis	0 (0.00)	1 (5.88)	1
Bronchial wall thickening	0 (0.00)	2 (11.76)	0.554
Pleural effusion	6 (42.86)	11 (64.71)	0.393
Hydropneumothorax	0 (0.00)	1 (5.88)	1

Subgroup analyses within the PJP + CMV cohort were exploratory. No multiplicity correction was applied to avoid type II error given the limited sample size, and findings should be interpreted with caution.

Bold values indicate mean statistical significance (*P* < 0.05).

**Table 5:** Cox regression analyses of risk factors for 28-day mortality in the PJP with CMV co-detection subgroup.

Parameter	Model 1		Model 2		Model 3	
	HR (95%CI)	*P*	HR (95%CI)	*P*	HR (95%CI)	*P*
Laboratory parameters						
BDG	1.176 (0.682, 2.028)	0.559	1.207 (0.715, 2.038)	0.481	1.279 (0.690, 2.371)	0.434
WBC	1.364 (0.560, 3.322)	0.495	1.277 (0.485, 3.363)	0.620	1.094 (0.401, 2.991)	0.860
NEU	2.71 (0.076, 97.075)	0.585	0.891 (0.025, 32.305)	0.950	0.454 (0.007, 30.777)	0.714
PaO₂	0.226 (0.032, 1.568)	0.132	0.189 (0.016, 2.297)	0.191	0.125 (0.007, 2.366)	0.166
PaCO₂	3.222 (0.174, 59.697)	0.432	0.765 (0.045, 13.132)	0.854	1.267 (0.062, 25.760)	0.878
PaO_2_/PaCO_2_	0.262 (0.053, 1.286)	0.099	0.414 (0.063, 2.708)	0.357	0.272 (0.030, 2.432)	0.244
LDH	1.427 (0.600, 3.393)	0.421	1.584 (0.538, 4.661)	0.404	1.566 (0.513, 4.780)	0.431
CRP	1.623 (0.975, 2.701)	0.063	**1.631 (1.040, 2.556)**	**0.033**	1.797 (0.988, 3.269)	0.055
IL-6	1.208 (0.921, 1.584)	0.171	**1.717 (1.109, 2.658)**	**0.015**	**1.915 (1.094, 3.352)**	**0.023**
PCT	**1.353 (1.061, 1.726)**	**0.015**	**1.419 (1.087, 1.852)**	**0.010**	**1.642 (1.171, 2.302)**	**0.004**
NT-proBNP	0.853 (0.569, 1.278)	0.441	0.876 (0.576, 1.334)	0.538	1.083 (0.590, 1.990)	0.796
LYM	0.991 (0.614, 1.600)	0.971	0.943 (0.473, 1.881)	0.868	1.026 (0.483, 2.182)	0.946
LYM-T	0.915 (0.663, 1.261)	0.586	0.716 (0.426, 1.205)	0.209	0.669 (0.377, 1.187)	0.169
LYM-B	0.886 (0.675, 1.163)	0.384	0.743 (0.493, 1.118)	0.154	0.764 (0.505, 1.156)	0.203
CD4+	0.943 (0.633, 1.405)	0.774	0.969 (0.580, 1.619)	0.906	0.976 (0.583, 1.635)	0.927
CD8+	1.114 (0.672, 1.847)	0.676	0.993 (0.476, 2.072)	0.984	0.892 (0.383, 2.078)	0.790
CD8+/CD3+	1.349 (0.493, 3.692)	0.561	0.785 (0.189, 3.262)	0.739	0.567 (0.110, 2.914)	0.497
CD4+/CD8+	0.775 (0.437, 1.374)	0.383	0.952 (0.463, 1.956)	0.893	1.046 (0.485, 2.255)	0.909
D-dimer	**2.415 (1.128, 5.166)**	**0.023**	**2.452 (1.246, 4.826)**	**0.009**	**4.686 (1.529, 14.358)**	**0.007**
Pulmonary imaging features						
Ground-glass opacity	0.848 (0.303, 2.374)	0.753	0.614 (0.157, 2.394)	0.482	0.844 (0.164, 4.332)	0.839
Other high-density opacities	1.107 (0.383, 3.202)	0.852	1.441 (0.416, 4.987)	0.564	1.655 (0.312, 8.769)	0.554
Nodules	0.341 (0.101, 1.153)	0.083	0.116 (0.02, 0.661)	0.015	0.012 (0.001, 0.246)	0.004
Consolidation	0.745 (0.186, 2.978)	0.677	0.724 (0.171, 3.059)	0.66	0.695 (0.162, 2.983)	0.624
Pulmonary bullae	0.904 (0.113, 7.235)	0.924	1.178 (0.119, 11.63)	0.889	1.595 (0.155, 16.401)	0.694
Cavities	0.725 (0.163, 3.227)	0.673	0.081 (0.007, 0.98)	0.048	0.029 (0.001, 0.59)	0.021
Pleural effusion	1.074 (0.381, 3.027)	0.893	1.367 (0.413, 4.531)	0.609	1.473 (0.410, 5.294)	0.553

Model 1: adjusted for age and sex; Model 2: additionally adjusted for history of diseases; Model 3: additionally adjusted for transplantation and immune agents combined with glucocorticoids use.

All continuous laboratory parameters were natural log-transformed prior to analysis. Hazard ratios (HR) correspond to a 1-unit increase in the log-transformed parameter.

Bold values indicate mean statistical significance (*P* < 0.05).

## 4 Discussion

*Pneumocystis jirovecii* pneumonia (PJP) is a well-recognized life-threatening opportunistic infection in immunocompromised patients, and its clinical burden has been increasingly shifting toward non-HIV populations in recent years (3, 16). Pulmonary cytomegalovirus (CMV) is frequently detected in patients with PJP, yet its clinical significance and prognostic impact remain largely undefined in non-HIV cohorts (17). In this retrospective study of 62 non-HIV patients with confirmed PJP who underwent bronchoalveolar lavage fluid (BALF) metagenomic next-generation sequencing (mNGS), we found that 50.0% had BALF CMV co-detection. Patients with CMV co-detection had significantly higher 28-day mortality and more severe dyspnea, along with distinct laboratory, imaging and co-pathogen profiles. IL-6, PCT and D-dimer were identified as independent prognostic factors for mortality within the PJP + CMV subgroup.

The 50.0% prevalence of BALF CMV co-detection in our cohort is consistent with the 54.3% reported in a previous retrospective study of non-HIV PJP patients, indicating that clinically relevant CMV co-detection is a common clinical observation rather than an incidental finding (17). In HIV-infected patients, earlier studies have yielded conflicting results regarding whether BALF CMV co-detection worsens PJP prognosis (5, 18), which may be partly explained by the relatively indolent course of PJP in the HIV population. In contrast, our data add to growing evidence that CMV co-detection is associated with substantially worse outcomes in non-HIV patients (17, 19). Notably, a very recent study of mechanically ventilated critically ill patients with PJP found that systemic CMV viremia was not an independent predictor of mortality after adjustment for acute respiratory distress syndrome and septic shock, suggesting that CMV may act primarily as a marker of disease severity in the most severely ill populations (4). The discrepancy between studies likely stems from differences in study populations and CMV diagnostic modalities: the prior study focused exclusively on intubated ICU patients and defined CMV by plasma viral load, whereas our cohort included a broader spectrum of non-HIV PJP patients and defined CMV via BALF mNGS detection. The significant mortality difference observed in our study indicates that BALF CMV co-detection carries important prognostic implications in the overall non-HIV PJP population. Notably, the primary outcome was all-cause 28-day mortality. We cannot attribute deaths directly to CMV infection or dual pathogen injury alone, as underlying diseases, treatment complications and other concurrent conditions may also have contributed. The excess mortality is likely driven by synergistic pulmonary injury from dual pathogens, compounded by the more abrupt and profound cellular immunosuppression that typically underlies PJP in non-HIV individuals (2, 16).

Three key laboratory findings merit further interpretation. First, serum (1,3)-β-D-glucan (BDG) levels were independently elevated in the PJP + CMV group after multivariable adjustment (20, 21). Together with the observed reduction in absolute total T lymphocyte counts, this association is biologically compatible with more profound cellular immune dysfunction in patients with BALF CMV co-detection. Such immune dysfunction could contribute to impaired host antifungal immunity, more severe PJP, and higher BDG levels; however, the retrospective observational design precludes determining the direction of these relationships or inferring that CMV reactivation caused lymphopenia or increased fungal burden. This interpretation is consistent with previous evidence that higher BDG levels correlate with greater disease severity in PJP. Clinically, markedly elevated BDG in a PJP patient should be interpreted in the context of overall disease severity and concurrent pathogen detection, rather than as evidence of a CMV-specific effect or fungal load alone. Second, the PJP + CMV group exhibited a distinct coagulation profile with elevated D-dimer and reduced fibrinogen, indicating dysregulation of the coagulation–fibrinolysis axis. This pattern aligns with the established link between systemic inflammatory response, endothelial injury and coagulation activation in severe pneumonia (7, 22, 23). CMV infection is known to exert indirect effects on host immunity and vascular endothelium, which can further amplify endothelial damage, promote a hypercoagulable state, and contribute to worse outcomes in patients with dual pathogen detection. The independent prognostic value of D-dimer in the PJP + CMV subgroup further supports the notion that coagulation dysfunction is a key pathway driving disease deterioration in this population (24–26). Third, the PJP + CMV group showed decreased absolute total T lymphocyte counts alongside a relative increase in CD3 + T cell percentage. This pattern reflects global lymphopenia with more severe depletion of non-T cell subsets, and is consistent with the paradigm that CMV reactivation occurs in the setting of profound cellular immunodeficiency. The reciprocal relationship between CMV reactivation and T-cell depletion forms a vicious cycle: severe immunosuppression predisposes patients to CMV reactivation, which in turn aggravates lymphopenia and further weakens host defense against PJP and other opportunistic pathogens (27–29).

Radiologically, strand-like or band-like shadows were nominally less frequent in the PJP + CMV group, but the difference did not survive correction for multiple comparisons. In clinical practice, the imaging manifestations of PJP and CMV pneumonia share extensive overlapping features, including diffuse ground-glass opacities, interstitial infiltrates, focal consolidations, and pleural effusions. While certain features such as nodular lesions and cavitations show a descriptive trend toward higher frequency with CMV co-detection, no pathognomonic radiological sign can reliably differentiate PJP with CMV co-detection from PJP alone (30, 31). This reinforces the critical role of pathogen detection in identifying concurrent CMV involvement. The unbiased nature of mNGS enables comprehensive profiling of the lower respiratory tract microbiome (10, 32, 33), and our data reveal that viral and fungal co-detections, rather than bacterial superinfection, predominate in patients with PJP and BALF CMV co-detection (34). Furthermore, a higher proportion of multiple co-detected pathogens was observed in non-survivors within the PJP + CMV subgroup, which supports the clinical value of broad-range pathogen screening for risk stratification (35).

From a clinical translation perspective, the combination of mNGS-based comprehensive pathogen screening and routine biomarker monitoring provides a practical strategy for the management of non-HIV PJP patients. On one hand, early mNGS testing of BALF can rapidly identify CMV and other opportunistic co-pathogens, avoiding missed diagnosis caused by targeted testing alone. On the other hand, dynamic monitoring of IL-6, PCT and D-dimer allows timely assessment of disease severity and risk of death, helping clinicians stratify high-risk patients and adjust the intensity of supportive care and anti-infective treatment accordingly.

A key interpretive caveat is the distinction between active infection and viral colonization or reactivation. As noted in previous studies, detection of CMV DNA in BALF does not equate to histologically proven CMV pneumonitis, and may simply represent viral shedding or reactivation in immunocompromised hosts (6, 36). Definitive diagnosis of invasive CMV pneumonia requires tissue biopsy, which is often infeasible in patients with severe hypoxemia. Nevertheless, the consistent association of CMV co-detection with distinct laboratory abnormalities and worse survival suggests that its presence carries clinical relevance regardless of its exact position on the infection–colonization continuum.

Several limitations of this study should be acknowledged. First, the single-center retrospective design with a relatively small sample size may introduce selection bias, as mNGS testing was ordered at the discretion of treating clinicians and may have been preferentially applied to more severely ill patients. Second, definitive pathological and quantitative pathogen evidence is lacking. CMV diagnosis was based on BALF mNGS detection combined with clinical and radiological correlation, rather than histopathological confirmation, which prevents definitive differentiation between invasive infection, viral reactivation and colonization. Meanwhile, quantitative *Pneumocystis jirovecii* fungal burden was not available, so we were unable to evaluate whether increased fungal load mediates the association between CMV co-detection and worse outcomes. Third, we did not perform multivariable adjustment for all-cause mortality in the full cohort due to limited sample size, and the independent prognostic value of BALF CMV co-detection warrants validation in larger cohorts. Fourth, detailed standardized quantification of immunosuppressive exposure was not available for stratified analysis. Our cohort included patients with highly heterogeneous underlying conditions (solid organ transplantation, autoimmune disorders, hematological malignancies), who received individualized immunosuppressive regimens varying in drug class, treatment course and dose intensity. Standardized unified records of cumulative steroid dosage and exact treatment duration were not uniformly archived across all electronic medical records, which prevented us from reliably extracting consistent quantitative exposure metrics for formal dose–response analysis or stratified sensitivity analysis. This is an inherent limitation of the retrospective single-center study design. Fifth, the 5-year enrollment period may carry temporal changes in mNGS detection protocols, ICU supportive care standards and anti-infective treatment strategies. Due to the limited sample size, we were unable to perform a stratified sensitivity analysis by enrollment year, and residual confounding by secular trends cannot be excluded. Sixth, the prognostic biomarker model was not validated in an independent external cohort, which limits the generalizability of our findings. Seventh, adjudicated cause of death was not uniformly and reliably available for all decedents due to the retrospective design, which precludes formal causal attribution of mortality to CMV co-detection or dual pathogen-related injury.

In conclusion, BALF CMV co-detection is present in approximately half of non-HIV patients with microbiologically confirmed PJP and is associated with higher unadjusted 28-day mortality. As CMV detection may represent viral reactivation rather than tissue-invasive pneumonia, and residual confounding by illness severity and immunosuppression cannot be excluded, these findings should be considered hypothesis-generating. IL-6, PCT and D-dimer show potential as exploratory prognostic markers in the co-detection subgroup, but their predictive value requires validation in larger, prospectively characterized cohorts. Nevertheless, these findings support the use of comprehensive mNGS-based pathogen screening in immunocompromised patients with suspected PJP, as identification of CMV co-detection carries important prognostic implications and may guide early risk stratification.

## Data Availability

The raw clinical and sequencing data supporting the findings of this study are not publicly available due to ethical restrictions and patient privacy protection related to human clinical and genomic information. De-identified aggregated data are presented within the manuscript and Supplementary materials. The underlying de-identified data are available from the corresponding author upon reasonable request, subject to approval by the institutional review board.
