# Supplementary material for: Clinical characteristics and prognostic factors in non-HIV patients with Pneumocystis jirovecii pneumonia and BALF cytomegalovirus co-detection: a retrospective cohort study
Source: Front Public Health. 2026 Aug 26;14:1921801. doi: 10.3389/fpubh.2026.1921801 (PMC13553864; doi:10.3389/fpubh.2026.1921801)
Supplement: Supplementary file 1 [file Table_1.DOCX]

**Table S1. Co-pathogens identified by mNGS stratified by survival status in the PJP group and the PJP with CMV co-infection group.**

| **Co-pathogens** | **PJP group (n=31)** | | **PJP+CMV group (n=31)** | |
| --- | --- | --- | --- | --- |
|  | **Survive (n=25)** | **Death (n=6)** | **Survive (n=14)** | **Death (n=17)** |
| **Bacteria** |  |  |  |  |
| *Enterococcus faecium* | 5 | 4 | 0 | 4 |
| *Acinetobacter baumannii* | 3 | 4 | 1 | 4 |
| *Pseudomonas aeruginosa* | 2 | 3 | 0 | 2 |
| *Stenotrophomonas maltophilia* | 3 | 3 | 0 | 3 |
| *Staphylococcus aureus* | 2 | 3 | 1 | 2 |
| *Klebsiella pneumoniae* | 2 | 2 | 1 | 2 |
| *Serratia marcescens* | 2 | 1 | 1 | 1 |
| *Proteus mirabilis* | 0 | 1 | 0 | 1 |
| *Elizabethkingia meningoseptica* | 0 | 1 | 0 | 1 |
| *Achromobacter xylosoxidans* | 0 | 1 | 0 | 1 |
| *Enterococcus faecalis* | 1 | 0 | 1 | 1 |
| *Bifidobacterium longum* | 1 | 0 | 0 | 0 |
| *Enterobacter cloacae complex* | 2 | 0 | 0 | 0 |
| *Streptococcus constellatus* | 1 | 0 | 0 | 0 |
| *Staphylococcus* spp. | 1 | 0 | 0 | 0 |
| *Tropheryma whipplei* | 2 | 0 | 0 | 0 |
| *Mycobacteria* spp. | 2 | 0 | 0 | 0 |
| *Escherichia coli* | 2 | 0 | 0 | 0 |
| **Fungi** |  |  |  |  |
| *Candida glabrata* | 2 | 2 | 2 | 2 |
| *Schizophyllum commune* | 1 | 0 | 0 | 0 |
| *Candida albicans* | 4 | 4 | 2 | 3 |
| *Aspergillus fumigatus* | 3 | 3 | 1 | 2 |
| *Lodderomyces elongisporus* | 0 | 2 | 0 | 0 |
| *Rhizomucor pusillus* | 0 | 1 | 0 | 1 |
| *Candida tropicalis* | 0 | 1 | 0 | 1 |
| *Aspergillus* spp. | 0 | 1 | 0 | 0 |
| *Aspergillus flavus* | 2 | 0 | 2 | 0 |
| *Trichosporon asahii* | 2 | 0 | 0 | 1 |
| *Candida lusitaniae* | 2 | 0 | 1 | 0 |
| **Viruses** |  |  |  |  |
| Herpes simplex virus 1 | 1 | 0 | 2 | 6 |
| Herpes simplex virus 2 | 1 | 0 | 0 | 0 |
| Varicella-zoster virus | 0 | 0 | 0 | 1 |
| Epstein-Barr virus | 3 | 1 | 10 | 4 |
| Human herpesvirus 6 (HHV-6) | 1 | 0 | 1 | 3 |
| Human herpesvirus 7 (HHV-7) | 3 | 0 | 5 | 2 |
| Torque teno virus | 14 | 6 | 4 | 6 |
| Torque teno mini virus | 2 | 1 | 0 | 1 |
| Molluscum contagiosum virus | 2 | 1 | 0 | 1 |
| Polyomavirus | 2 | 1 | 0 | 1 |
| Human adenovirus | 2 | 0 | 3 | 0 |
| Coronavirus | 2 | 0 | 0 | 0 |

Data are presented as number of patients in whom the pathogen was detected. CMV is excluded as it served as the grouping variable. One patient may harbor multiple co-pathogens. 0 indicates no detection of the corresponding pathogen in the subgroup. PJP, Pneumocystis jirovecii pneumonia; CMV, cytomegalovirus; mNGS, metagenomic next-generation sequencing.
