# Supplementary material for: Clinical characteristics and prognostic factors in non-HIV patients with Pneumocystis jirovecii pneumonia and BALF cytomegalovirus co-detection: a retrospective cohort study
Source: Front Public Health. 2026 Aug 26;14:1921801. doi: 10.3389/fpubh.2026.1921801 (PMC13553864; doi:10.3389/fpubh.2026.1921801)
Supplement: Supplementary file 2 [file Table_2.DOCX]

**Supplementary Methods:**

**mNGS Sequencing and Quality Control**

Sequencing parameters. Sequencing was performed with 75 cycles of single-end reads on the Illumina NextseqCN500 platform.

Controls. Peripheral blood mononuclear cells (PBMCs) at 10⁵ cells/mL from healthy donors were processed using the same extraction and library preparation protocol as the negative control. Sterile deionized water was processed identically as the non-template control (NTC).

Quality filtering. Low-quality reads (Phred score <20), adapter contaminations, duplicate reads, and reads shorter than 50 bp were removed using Trimmomatic. Reads with low sequence complexity were filtered using Kcomplexity with default parameters.

Penalty thresholds. For species-level detection, a 5% penalty was applied; for genus-level detection, a 10% penalty was applied.
